# CYPA, a novel and potential genetic adjuvant enhanced HIV-1 DNA vaccine immunoreactivity

**DOI:** 10.1186/1742-4690-9-S2-P3

**Published:** 2012-09-13

**Authors:** J Hou, Y Liu, Y Shao

**Affiliations:** 1 China CDC, Beijing, China

## Background

Recent research highlight that newly synthesized HIV-1 capsid protein is required to induce dendritic cell activation through a pathway involving interaction with peptidyl-prolyl cis-trans isomerase cyclophilin A (CYPA),which subsequently induce an antiviral type I interferon response and activation of T cells.

Previous studies also revealed HIV Gag-CYPA interaction inhibited recognition by host restriction factors. In another perspective, the inhibition effect might be benefit for HIV Gag immunogenicity.

Consequently, we assume CYPA might be a potential genetic adjuvant co-inoculation with HIV-1 Gag DNA vaccine, which not only circumvent the restriction of host factors, but also activate dendritic cell and enhance adaptive immune response.

## Methods

In this study, 6-8-week-old female BALB/C mice were administrated thrice intramuscularly with pDRVI4.0-Gag HIV-1 DNA vaccine co-formulated with pDRVI4.0-CYPA plasmid at two weeks interval. IFN-γ ELISPOT, Gag-specific ELISA, IgG isotype ELISA detected cellular and humoral immunological response.

## Results

The regimen of HIV-1 Gag DNA premixed with CYPA DNA induced robust immune responses in mouse model. IFN-γ ELISPOT result demonstrated Gag DNA alone only generated 180 ± 63(mean ± SD) SFCs/million. However, Gag DNA combined with CYPA DNA strategy produced 353 ± 80(mean ± SD) SFCs/million. In humoral immune response, Gag DNA co-inoculation with CYPA DNA showed high level antibody titer (GMT=22800),whereas Gag DNA alone induced slight antibody response (GMT=8000). IgG isotype results confirmed that co-vaccination with CYPA DNA induced Th1-bias immune response, however, Gag DNA alone activated Th1/Th2 response in balance.

## Conclusion

This is the first report demonstrating that mixture of CYPA DNA and HIV-1 gag DNA vaccine could induce robust cellular and humoral immune response in the mouse model. Ongoing studies are focusing on construction of dual expression cassette DNA vaccine, which including both Gag and CYPA gene. Further research will also construct others HIV DNA vaccine involving human or non-human primates CYPA.

